# Mapping the relationship between flow experience and music performance anxiety: a scoping review

**DOI:** 10.3389/fpsyg.2026.1746749

**Published:** 2026-02-25

**Authors:** Nery Borges, Helena Marinho, Marcos Araújo, Anabela Pereira, Sofia Serra

**Affiliations:** 1 Department of Communication and Arts, Institute of Ethnomusicology – Center for Studies in Music and Dance (INET-md), University of Aveiro, Aveiro, Portugal; 2 Instituto de Artes, Programa de Pós-Graduação em Música, Universidade Federal do Rio Grande do Sul (UFRGS), Porto Alegre, Rio Grande do Sul, Brazil; 3 Department of Psychology, School of Social Sciences, Center for Research in Education and Psychology (CIEP), University of Évora, Évora, Portugal

**Keywords:** flow experience, flow experience–music performance anxiety association, music performance anxiety, musicians, scoping review

## Abstract

**Introduction:**

This scoping review systematically mapped and synthesized the literature examining the relationship between Flow Experience (FE) and Music Performance Anxiety (MPA) in musical performance and educational contexts. Examining their interaction is crucial to understand how musicians balance emotional activation and attentional focus to achieve optimal performance states.

**Methods:**

Following PRISMA-ScR, a comprehensive search across Scopus, Web of Science, PubMed, PsycINFO, and SAGE databases identified 18 empirical studies. These were systematically analyzed according to author(s) and year, main aim, sample characteristics, measurement instruments, study design, FE–MPA interaction, mediating or moderating factors, performance setting, analytical or procedural approach and main findings.

**Results:**

Findings reveal a predominant negative correlation between FE and MPA across dispositional, situational, and psychophysiological measures. However, moderate anxiety levels can coexist with or even facilitate flow when they are interpreted as a challenge rather than a threat. Emotional self-regulation, self-efficacy, and self-esteem emerged as key mediating factors, while interventions involving yoga, mindfulness, and self-regulation training consistently reduced MPA and enhanced FE propensity.

**Discussion:**

Overall, the findings highlight a dynamic continuum between FE and MPA, suggesting that anxiety regulation is not merely inhibitory but can function as a potential precursor to flow. Despite substantial empirical work examining FE and MPA as individual constructs, the present synthesis demonstrates that an integrated understanding of their interaction remains methodologically fragmented. Future research should therefore prioritize multimodal and longitudinal designs to clarify underlying mechanisms and support the development of evidence-informed approaches to performance preparation and emotional regulation in musicians.

## 1 Introduction

Public musical performance elicits a range of psychophysiological reactions, spanning from adaptive to maladaptive responses (Kenny and Osborne, 2006; Cohen and Bodner, 2021). These reactions are influenced by numerous contextual, psychological, and cognitive variables, ultimately shaping the performers’ subjective evaluations of their performance experience as either positive or negative (Clark et al., 2014). Among these, Music Performance Anxiety (MPA) and Flow Experience (FE) stand out as two distinct phenomena that can directly affect both the quality of performances and the longevity of performers’ careers. High MPA levels can have a negative impact on both music students and professional musicians, regardless of their instrument, role, or type of performative activity, sometimes leading to career abandonment (Kenny, 2011; Spahn, 2015; Kaleńska-Rodzaj, 2018). Conversely, the Flow Experience (FE) is widely recognized as a highly motivating phenomenon, characterized by total immersion during a performance activity, followed by feelings of satisfaction and well-being upon its completion (Csikszentmihalyi, 1990; Nakamura and Csikszentmihalyi, 2002). This contrast leads to the suggestion of an antithetical relationship between the two phenomena (Kirchner, 2011), with FE correlating positively with performance experiences and well-being, and excessive MPA correlating negatively with both.

Nevertheless, several authors have pointed out that this relationship may not be strictly antagonistic. Emerging perspectives indicate that controlled levels of MPA can manifest both facilitating and debilitating effects, depending on how it is appraised and regulated by the performer (Papageorgi et al., 2007; Papageorgi et al., 2013; Papageorgi, 2021). Moderate levels of arousal may sustain energy, attention, and motivation, whereas excessive activation leads to cognitive interference and self-criticism, impairing performance (Osborne and Kenny, 2008; Kenny, 2011). This duality suggests that MPA and FE can coexist under specific conditions, especially when the performer perceives the challenge–skill balance as optimal (Lamont, 2012; Antonini Philippe et al., 2022a).

Building on this complexity, empirical and theoretical research has increasingly addressed this interaction, aiming to understand how FE can help regulate MPA and, conversely, how anxiety may modulate flow propensity (Kirchner et al., 2008; Fullagar et al., 2013; Cohen and Bodner, 2019a; Moral-Bofill et al., 2022). However, despite the growing interest, there has been no systematic effort to integrate and map the evidence on how both constructs have been studied together across different samples, methodologies, and performance contexts.

To address this identified gap, the present review systematically maps and synthesizes the scientific literature that has examined FE and MPA together, identifying how these constructs have been conceptualized, measured, and interconnected across different musical and educational contexts. By integrating diverse theoretical and methodological perspectives, this review provides a comprehensive overview of current evidence, highlighting conceptual tendencies, methodological diversity, and empirical gaps that may guide future research and inform the development of interventions promoting optimal engagement and well-regulated MPA, supporting emotional balance and self-regulation in musicians.

### 1.1 Flow experience

The FE concept was defined by Csikszentmihalyi (1975), who described it as “the way people describe their state of mind when consciousness is harmoniously ordered, and they want to pursue whatever they are doing for its own sake” (Csikszentmihalyi, 1990, p. 6). Csikszentmihalyi (1990) observed that individuals experiencing this mental state when performing tasks often use the metaphor of being carried effortlessly by a current and thus adopted the designation of “flow.” As stated by Nakamura and Csikszentmihalyi (2002), this subjective state, which is experienced when individuals are completely absorbed by an activity, presents, as its main characteristics, concentration exclusively on the task in hand without any type of distraction, associated with satisfaction and a feeling of well-being. Therefore, flow theory is connected to the concept of optimal experience. According to Csikszentmihalyi (1990), FE displays 9 indicators: (1) challenge-skill balance; (2) clear goals; (3) unambiguous feedback; (4) concentration on the task at hand; (5) sense of control; (6) loss of self-consciousness; (7) merging action and awareness; (8) time transformation; and (9) autotelic experience. Nakamura and Csikszentmihalyi (2009, p. 196) divide these indicators into two groups: conditions (indicators 1, 2, and 3) and characteristics (indicators 4, 5, 6, 7, 8, and 9). Although a substantial body of research has operationalized flow using its nine core dimensions, recent work has increasingly problematised how this construct is defined and measured in empirical studies.

Recent work has highlighted that variability in flow findings is closely associated with how the construct is operationalized and measured (Bricteux et al., 2017; Abuhamdeh, 2020; Moneta, 2021). In this context, operationalization refers to the specific measurement assumptions, instruments, and analytical procedures used to translate the theoretical construct of flow into empirical indicators. Prior work has shown that differing operational assumptions—such as the use of continuous versus episodic measurement approaches—are associated with divergent empirical patterns of flow-related outcomes (Bricteux et al., 2017; Abuhamdeh, 2020). Within this methodological background, research on flow has expanded across multiple applied domains, including musical performance.

As claimed by Araújo (2016), flow has been studied in various fields since the 1980s (sport, work, and leisure); however, in the field of music, empirical research has only gained ground since 2000. Flow has been studied in musicians with different levels of experience and in various contexts: live performances (Wrigley and Emmerson, 2013; Spahn et al., 2021), group contexts (Kraus, 2003; Sawyer, 2006; Bishop, 2018), musical practice (Waite and Diaz, 2012), jazz performance (Sawyer, 2006; Hart and Di Blasi, 2015), practice efficiency, self-efficacy and self-regulation (Miksza and Tan, 2015), and self-regulatory practices and flow in highly specialized musicians (Araújo and Hein, 2016). Flow also positively relates to the well-being of music students and performance quality (Fritz and Avsec, 2007; Moral-Bofill et al., 2022), and daily practice time (Waite and Diaz, 2012; Marin and Bhattacharya, 2013; Heller et al., 2015).

Studies have established a positive association between FE and non-cognitive factors, including trait emotional intelligence and practice intensity (Marin and Bhattacharya, 2013), as well as intrinsic motivation and openness to experience (Butkovic et al., 2015), and personality traits such as higher conscientiousness and lower neuroticism (greater emotional stability) (Ullén et al., 2012). In musicians, FE has also been associated with higher extraversion (Heller et al., 2015). In addition, studies have addressed the relationship between FE and physiological measures such as heart rate, blood pressure, heart rate variability, activity of the zygomaticus major muscle and respiratory depth, reporting significant associations between state flow ratings and cardiovascular/autonomic indices, alongside deeper respiration and greater zygomaticus major activity during higher-flow performances (De Manzano et al., 2010; Harmat et al., 2015). Related work has also linked specific flow dimensions to autonomic balance/regulation during performance (Thomson and Jaque, 2012; Jaque et al., 2020). The identification of predictors for the flow experience has also been researched, with recurring predictors/facilitators including self-confidence/self-belief while playing and the motivation to experience and express emotions through music (Bloom and Skutnick-Henley, 2005; Spahn et al., 2021), as well as the identification and feasibility of strategies to promote FE during performance. Several literature reviews have focused on flow in musical contexts (Chirico et al., 2015; Kim, 2016; Keay, 2018; Tan and Sin, 2021). In recent years, narrative reviews and thematic collections have contributed to updating theoretical, methodological, and educational perspectives on flow in musical performance (Antonini Philippe et al., 2022a; Biasutti and Habe, 2023; Habe and Biasutti, 2024). None of these publications, however, studied the correlation between FE and MPA.

### 1.2 Music performance anxiety

Kenny (2011) defined MPA as a marked and persistent anxious perception experience associated with musical performance. MPA has been associated with biological and/or psychological vulnerabilities and/or experiences that cause anxiety, and is expressed through combinations of affective, cognitive, somatic, and behavioral symptoms. MPA can be focal, occurring only in association with musical performance, or associated with disorders such as social phobia (Valentine, 2004; Kenny, 2011). Artists affected by MPA may experience symptoms such cardiovascular and autonomic changes (e.g., rapid heart rate, cardiovascular changes, dry mouth), cognitive changes (with impaired attention and concentration and consequent memory failure), respiratory changes, including shortness of breath, tachypnoea and sensations of suffocation (Hiner et al., 1987; Wesner et al., 1990), digestive changes, and psychological symptoms, including insecurity, apprehension, nervousness, anguish, crying and fright, as well as fatigue, initial insomnia or sleep problems, nightmares, and feelings of depersonalization or derealization (Gentil and Gentil, 2012).

High levels of MPA can bring about negative stress reaction symptoms (Fernholz et al., 2019; Guyon et al., 2020b), compromising public performance skills, regardless of instrument (Ryan and Andrews, 2009) and professional experience (Kenny et al., 2014). MPA is one of the most frequently reported multidimensional conditions among musicians, often involving significant difficulties, with an estimated prevalence rate of between 16.5 and 60% (Fernholz et al., 2019). Barros et al. (2022) reported values between 16 and 83.1% as regards the prevalence of MPA in studies with music students. The personal exposure that induces the occurrence of high levels of MPA can be linked to high professional, physical and psychological demands that are not always met, expectations during the performance (LeBlanc, 1994; Papageorgi et al., 2007), and vulnerability to factors such as the size or composition of the audience and external evaluation (LeBlanc, 1994; Spahn, 2015; Osborne and Kirsner, 2022), in many cases triggering chronically debilitating impacts (Kenny and Osborne, 2006). Stage fright and failure, lack of concentration and distraction, and impaired motor skills can directly affect the musician’s career and professional future (Rocha et al., 2011; Studer et al., 2011; Reitano, 2013). MPA can accompany musicians throughout their lives but does not always affect the quality of the performance (Valentine, 2004; Kenny, 2011). According to Papageorgi (2021), coping strategies for MPA are decisive; a positive perception of the performance can reduce the detrimental symptoms of (adaptive) MPA, and, when negative, can intensify (maladaptive) MPA.

MPA has been linked to multiple contextual and individual factors, particularly solo performance and concert settings (Papageorgi et al., 2010; Studer et al., 2012, 2014; Orejudo et al., 2017; Robson and Kenny, 2017; Casanova et al., 2018; Sulun et al., 2018; Papageorgi et al., 2010; Studer et al., 2012, 2014; Orejudo et al., 2017; Robson and Kenny, 2017; Casanova et al., 2018; Sulun et al., 2018). Social-perceptual factors, such as low self-confidence and low self-esteem, are also associated with higher MPA levels (Liston et al., 2003; Miller and Chesky, 2004; Kirchner et al., 2008). In addition, individual variables such as self-punishment (Alzugaray et al., 2016), perfectionism (Sulun et al., 2018; Dobos et al., 2019) low optimism and reduced self-efficacy (Alzugaray et al., 2016; Orejudo et al., 2017; Robson and Kenny, 2017; Paliaukiene et al., 2018), as well as high trait anxiety and total anxiety (Ioannou et al., 2018; Umuzdaş et al., 2019), have been identified as relevant contributors to heightened MPA. Finally, some studies report significantly higher levels of MPA among women compared to men (Yondem, 2007; Iusca and Dafinoiu, 2012; Orejudo et al., 2017; Sulun et al., 2018; Dobos et al., 2019). Additional information can also be found in systematic and narrative reviews on MPA addressing its prevalence and epidemiological characteristics (Brugués, 2011; Fernholz et al., 2019; Barros et al., 2022), its conceptual framing and associated individual and contextual factors (Taborsky, 2007; Fernholz et al., 2019; Lima et al., 2024), as well as evidence regarding coping strategies and therapeutic or intervention approaches (Kenny, 2005; Nagel, 2010; Burin and Osório, 2016; Gómez-López and Sánchez-Cabrero, 2023; Bakhtiari et al., 2025; Kinney et al., 2025; Nicholl and Abbott, 2025).

### 1.3 Rationale and aim of the review

Despite the growing body of research on FE and MPA, most empirical and theoretical studies have investigated these constructs separately, addressing distinct samples, performance settings, and methodological paradigms. This fragmentation has limited an integrated understanding of how both experiences have been jointly conceptualized, measured, and contextualized in the field of musical performance. Moreover, existing studies conceptualize the relationship between FE and MPA in divergent ways. While some approaches frame the constructs as antagonistic, suggesting that elevated anxiety constrains flow, others indicate that moderate and adaptive levels of performance-related arousal may coexist with, or even facilitate, flow. Additional perspectives propose indirect relationships, in which psychological and contextual variables such as self-efficacy, emotion regulation, and performance conditions mediate or moderate the FE–MPA dynamic. This plurality of conceptualisations, combined with heterogeneous methodological approaches, has hindered a coherent synthesis of how both constructs relate within musical performance contexts.

Given the heterogeneity of research approaches and the lack of synthesis regarding shared frameworks and assessment strategies, a scoping review design was considered appropriate. Accordingly, this review was guided by the following research question: How have FE and MPA been jointly conceptualized, measured, and interpreted among musicians and music students within performative and educational contexts? To address this question, the review aims to systematically map and synthesize the literature that has examined FE and MPA together, identifying how these constructs have been studied in relation to each other across theoretical, methodological, and instrumental dimensions. The purpose is not to determine causal or statistical relationships, but rather to clarify how the scientific literature has defined, measured, and connected both constructs in performative and educational musical contexts. A preliminary search for existing scoping and systematic reviews on the topic was conducted in accordance with the methodological guidance outlined by Peters et al. (2020). No previous reviews addressing the relationship between FE and MPA were identified, reinforcing the need for this mapping effort. By doing so, this review provides an integrative overview of the current state of knowledge, highlighting conceptual tendencies, methodological diversity, and empirical gaps that may inform future research and guide the design of interventions promoting both optimal experience and adaptive anxiety regulation in musicians.

## 2 Method

This study was conducted as a scoping review, following the methodological guidelines of the PRISMA-ScR (Tricco et al., 2018) and the JBI Manual for Evidence Synthesis (Peters et al., 2020). In this framework, musicians and music students were defined as the sample, Flow Experience (FE) and Music Performance Anxiety (MPA) as the central concepts, and musical performance situations within educational or professional contexts as the context. This approach allowed for the inclusion of diverse study designs and heterogeneous evidence, enabling a systematic mapping of how FE and MPA have been conceptualized, measured, and investigated across different settings.

The literature search was conducted across Scopus, Web of Science, PubMed, PsycINFO (EBSCOhost), SAGE, using the Boolean string (“flow” OR “flow experience” OR “flow state” OR “optimal experience”) AND (“music performance anxiety” OR “MPA” OR “musician anxiety” OR “musical performance anxiety” OR “performance anxiety”) AND (“musician*” OR “music student*” OR “performer*” OR “music performance”) AND (“relationship” OR “correlation” OR “association” OR “relation”). All records were screened following PRISMA-ScR procedures, ensuring transparency and reproducibility throughout all stages of the review.

The inclusion criteria were: (1) publications dated between 1975 and September 2025, covering the period from Csikszentmihalyi’s *Beyond boredom and anxiety: Experiencing flow in work and play* (1975) up to September 2025, date of final search execution; (2) articles published in English; (3) studies conducted within the domain of music performance, involving students, professional musicians, and educational or performative contexts; (4) publications that examined both FE and MPA or analyzed their interrelations; and (5) studies using validated measurement tools or empirical indicators for both constructs; and (6) peer-reviewed journal articles only, to ensure methodological consistency and data reliability, even though the PRISMA-ScR framework encourages the inclusion of gray literature to ensure comprehensive coverage.

Exclusion criteria comprised: (1) studies addressing only one of the constructs (FE or MPA); (2) research outside the domain of music performance or unrelated to performative or educational contexts; (3) studies lacking validated measurement tools for both constructs; (4) gray literature such as thesis, dissertations, conference abstracts, or non-peer-reviewed publications; (5) retracted articles; and (6) literature reviews or meta-analyses.

To address risk of bias, the included studies were critically appraised independently by two reviewers. Any disagreements were resolved through discussion and, when necessary, consultation with a third reviewer, following the guidance of the *JBI Manual for Evidence Synthesis* (Peters et al., 2020).

Data extraction was conducted using a structured Microsoft Excel spreadsheet that compiled key information from each included study, such as authors, year of publication, journal, country, aims, study design, and sample characteristics (number of participants, gender distribution, educational or professional level, instrument, and site of data collection). In accordance with the methodological guidance of Peters et al. (2020), the organization of the extracted data prioritized transparency in data structuring, coherence between the research question and the mapping strategy, and the use of clear and reproducible analytical categories. The extracted information was organized to identify who was studied, what was examined, and under which conditions the studies were conducted, including participant profiles, research aims, FE–MPA interaction types, measurement instruments, mediating or moderating factors, performance settings, and analytical or procedural approaches. This approach enabled a systematic and transparent mapping of how FE and MPA have been investigated across different samples, research foci, and contexts, and supported the construction of Table 1 as well as the development of the thematic categories used to organize the Results section. This scoping review protocol was preregistered in the Open Science Framework (Borges et al., 2025).

**Table 1:** Summary of empirical studies included in the scoping review on the relationship between FE and MPA.

Author(s) and year	Main aim	Sample characteristics	Measurement instruments	Study design	FE – MPA Interaction	Mediating/moderating factors	Performance setting	Analytical or procedural approach	Main findings
Khalsa and Cope (2006)	To evaluate the effects of a yoga-based intervention on musicians’ FE and MPA	*N* = 18 (♀ 9, ♂9); semi-professional musicians; United States	FE: DFS-2 (dispositional); MPA: PAQ (state)	Quasi-experimental	Direct	Mood state, musculoskeletal disorders: POMS, PRMD	Real; educational intervention; summer program	Yoga-based intervention (8 weeks); pre–post measures of FE and MPA; *t*-tests and correlations	Yoga intervention reduced MPA significantly; slight, non-significant FE increase
Kirchner et al. (2008)	To examine the relationship between FE propensity and MPA	*N* = 90 (♀ 52, ♂ 38); university music students; United States	FE: MFS (dispositional); MPA: PAI (trait)	Cross-sectional	Direct	Destructive self-criticism, attentional focus, self-confidence, arousal level, age, experience, performance condition	Educational; rehearsal; professional orchestra	Questionnaires on self-efficacy, FE, and MPA; Pearson correlations and multiple regressions	Higher FE scores were associated with lower MPA and reduced self-criticism
Lamont (2012)	To explore musicians’ emotional experiences, focusing on the coexistence of FE and MPA	*N* = 35 (♀ 27, ♂ 8); music and psychology students; UK	SEM-DS; state FE – state MPA	Qualitative	Indirect coexistence	Motivation, challenge–skill balance, destructive self-criticism, attentional focus, self-confidence, arousal level, age, experience, performance context	Real; performance; university	Semi-structured interviews; thematic analysis of musicians’ narratives on coexisting FE and MPA	FE and MPA coexisted; optimal challenge–skill balance allowed MPA to precede FE
Fullagar et al. (2013)	To assess challenge–skill balance, FE, and MPA during performance preparation	*N* = 27 (♀ 13, ♂ 14); university music students; USA	FSS (adapted; state); MPA: self-reported anxiety levels (state)	Longitudinal	Direct	Challenge–skill balance	Educational; exam performances	Weekly measures of FE, affect, and MPA (7 weeks); longitudinal repeated-measures analysis	FE increased as MPA decreased; challenge–skill balance predicted optimal experience
Butzer et al. (2016)	To examine the effects of yoga training on FE, mindfulness, and performance MPA in professional musicians	*N* = 60 (♀ 35, ♂ 25); professional musicians; USA	FE: DFS-2 (dispositional); MPA: PAQ (trait)	Quasi-experimental	Direct	Mindfulness, mood state (FFMQ, POMS)	Real; educational intervention; summer program	Yoga training (8 weeks); pre–post assessments; ANOVA and paired *t*-tests	Yoga enhanced FE and mindfulness while reducing MPA
Thomson et al. (2017)	To evaluate the impact of intensive opera training on FE and MPA	N = 123 (♀ 104, ♂ 19); professional and pre-professional singers; USA	FE: DFS-2 (dispositional); MPA: STAI-Y2 (trait)	Experimental	Direct	Emotion regulation difficulties, perfectionism, internalized shame: DERS, MPS, ISS	Real and rehearsal performance; educational	Opera training (2–4 weeks). Observation of live performances; correlational analysis of FE and MPA (pre–post measures)	FE increased; MPA decreased; evidence for mutual inhibition between constructs
Cohen and Bodner (2019a)	To test the effects of a performance-skills intervention on FE and MPA among music therapy students	*N* = 24 (♀ 10, ♂ 14); grad music therapy students; Israel	FE: DFS-2 (short/full) (dispositional); MPA: PAI, STAI, SPA (trait)	Experimental	Direct	Self-efficacy, positive affect, psychological distress performance quality, affective state: BSI, PQ, PANAS	Simulated; educational; self-regulation training.	Self-regulation training; pre–post design; t-tests and qualitative feedback analysis	Intervention reduced MPA and strengthened negative correlation between FE and MPA
Cohen and Bodner (2019b)	To investigate the relationship between FE frequency and MPA, and the moderating effect of emotional contagion	*N* = 202 (♀ 100, ♂ 102); orchestral musicians; Israel	FE: DFS-2 (short) (dispositional); MPA: PAI (trait)	Cross-sectional	Direct + moderated	Musical emotional contagion: MEC	Professional orchestra; rehearsal	FE, MPA, and emotional contagion scales; hierarchical regression analysis	FE and MPA were negatively correlated; emotional contagion moderated this association
Cohen and Bodner (2021)	To explore background and contextual variables influencing FE and MPA	*N* = 202 (♀ 100, ♂ 102); orchestral musicians; Israel	FE: DFS-2 (short; dispositional); MPA: PAI (trait)	Cross-sectional	Direct + contextual moderation	Age, experience, instrument	Professional orchestra; rehearsal.	FE, self-efficacy, and MPA questionnaires; correlation and regression analyses	FE negatively correlated with MPA; older and more experienced musicians reported higher FE
Tan et al. (2021)	To examine how grit and growth mindset relate to dispositional Flow and MPA	*N* = 162 (♀ 59, ♂ 103); adult musicians; multinational	FE: DFS-2 (dispositional); MPA: MPAI-A (trait)	Cross-sectional	Direct + mediated	Grit, perseverance growth mindset, personality traits, musical sophistication: TIPI, Gold-MSI, MS, GS	General musical activity	Grit, growth mindset, and flow questionnaires; correlational and regression analyses	FE was negatively correlated with MPA; non-cognitive traits promoted higher FE levels
Spahn et al. (2021)	To compare FE and MPA before, during, and after live performance	N = 363 (♀ 50.4%, ♂ 49.6%); classical musicians; Germany	FE: FSS (short; state); MPA: PQM (state)	Cross-sectional	Direct (time-based)	Self-efficacy, motivation	Live performance; professional orchestras	Pre/during/post-concert assessment of FE and MPA; paired *t*-tests	FE and MPA negatively correlated across performance phases
Guyon et al. (2022)	To assess the influence of audience presence on FE dimensions and MPA	*N* = 121 (♀ 69, ♂ 52); music students; Switzerland	FE: FSS-2 (French; state) MPA: STAI (French; state), CSAI-2R, K-MPAI-R	Cross-sectional	Direct (within-subject)	Audience presence, affect, depression, psychopathology, performance quality: PHQ-9, BDI-2, LSAS, MQP, PMPTQ, SAMq	Private vs. public performance.	Public vs. private performance conditions; FE and MPA measures; mixed-design ANOVA	Higher MPA decreased specific FE dimensions, including focus and control
Jha et al. (2022)	To investigate psychophysiological correlates of FE and MPA using heart rate variability (HRV)	*N* = 22 (♀ 13, ♂ 9); piano students; Canada	FE: FSS (state); MPA: HADS (state)	Cross-sectional	Direct + physiological	HRV indices	Laboratory; piano solo performance	HRV recording pre/during/post-performance + FSS; correlation and multiple regression analyses	Negative FE–MPA correlation; higher vagal modulation → greater flow
Moral-Bofill et al. (2022)	To evaluate the effects of a self-regulation training program on FE and MPA	*N* = 62 (♀ 42, ♂ 20); conservatoire students and teachers; Spain	FE: EFIM (state); MPA: K-MPAI (trait + state)	Quasi-experimental	Direct (pre–post)	Self-regulation, social skills: SSS	Conservatoire; Online	Self-regulation program (12 weeks); repeated-measures ANOVA	Post-test results showed increased FE and stronger negative FE–MPA correlation
Rakei et al. (2022)	To explore relationships between FE, MPA, emotional intelligence, and personality traits	*N* = 664 (♀ 186, ♂ 469); contemporary musicians; multinational	FE: DFS-2 (dispositional); MPA: STAI-Y2 (trait)	Cross-sectional	Direct + moderated	Emotional intelligence, musical sophistication personality: IPC, TEIQue-SF, Gold-MSI, MS, GS	Online survey; orchestral musician.	FE, MPA, self-esteem, and emotional intelligence; path analysis	FE positively correlated with emotional intelligence and negatively with MPA
Antonini Philippe et al. (2022b)	To explore factors promoting FE and reducing MPA in experienced musicians	*N* = 11 (♀ 6, ♂ 5); professional & student musicians; Switzerland	Graphs + interviews; state FE – state MPA	Qualitative	Indirect (qualitative interpretation)	Self-confidence, preparation, social support	Retrospective performance; Interviews	Elicitation interviews; interpretative phenomenological analysis (IPA)	Self-awareness, preparation, and social support enhanced FE and reduced MPA
Jiang and Tong (2024)	To examine effects of psychological capital (PsyCap) on MPA through FE and self-esteem	*N* = 329 (♀192, ♂ 137); undergraduate students; China	FE: FSS-2 (adapted; state); MPA: K-MPAI (trait + state)	Cross-sectional	Mediated	Psychological capital, self-esteem: PsyCap, RSES	Electronic after performance; examination; three university conservatories	Psychological capital, self-esteem, FE, and MPA scales; structural equation modeling (SEM)	Higher PsyCap increased FE and self-esteem, which together reduced MPA

The table details the author(s) and year, main aim, sample characteristics, measurement instruments, study design, FE–MPA interaction, mediating or moderating factors, performance setting, analytical or procedural approach and main findings.

Note to interactions column: *Direct* refers to a quantitative relationship between Flow Experience (FE) and Music Performance Anxiety (MPA) tested statistically (e.g., correlation, regression, or pre–post comparison). *Conceptual* designates theoretical or non-empirical approaches proposing an antagonistic or inverse link between the two constructs. *Moderated* and *Contextual moderation* indicate that the FE–MPA relationship varies according to specific moderating or contextual factors, such as emotional contagion, age, experience, or type of performance. *Mediated* refers to an indirect relationship in which a third variable (e.g., self-esteem, psychological capital) explains or transmits part of the association between FE and MPA. *Physiological* denotes studies incorporating psychophysiological indicators, such as heart rate variability, to infer changes related to FE and MPA. *Indirect* refers to qualitative or inferred relationships not tested statistically, often derived from participants’ narratives or interpretive analyses.

MPA instruments: Performance Anxiety Inventory (PAI; Montello et al., 1990), the Hospital Anxiety and Depression Survey (HADS; Zigmond and Snaith, 1983), the Kenny Music Performance Anxiety Inventory (K-MPAI; Kenny, 2009), Kenny Music Performance Anxiety Inventory–Revised (K-MPAI-R; Antonini Philippe et al., 2022b), the Music Performance Anxiety Inventory for Adolescents (MPAI-A; Osborne et al., 2005), the State–Trait Anxiety Inventory (STAI-Y; Spielberger, 1983b), the Trait Anxiety Inventory (STAI-Y2, Spielberger, 1983a), the Signs of Performance Anxiety (SPA; based on Montello et al., 1990), the Performance Anxiety Questionnaire (PAQ; Cox and Kenardy, 1993), the Fragebogen zum Auftritt für MusikerInnen – Performance-specific Questionnaire on Music Performance Anxiety (FZAM/PQM; Spahn et al., 2016), the Competitive State Anxiety Inventory–2 Revised (CSAI-2R; Cox et al., 2003).

FE instruments: Flow State Scale (FSS; Jackson and Marsh, 1996), the Dispositional Flow Scale-2 (DFS-2; Jackson and Eklund, 2002, 2004), the Short Dispositional Flow Scale-2 (DFS-2-Short; Jackson et al., 2008), the Flow State Scale for Musical Performers (EFIM; Moral-Bofill et al., 2022), the Flow Short-Scale – Global Flow Score (FSS; Rheinberg et al., 2003), the Music in Flow Survey (MFS; Bloom and Skutnick-Henley, 2005), Strong Experiences with Music – Descriptive System (SEM-DS; Gabrielsson and Wik, 2003).

Mediating/moderating instruments: Five Facet Mindfulness Questionnaire (FFMQ; Baer et al., 2008), the Internality, Powerful Others and Chance (IPC; Levenson, 1981), the Trait Emotional Intelligence Questionnaire (TEIQue-SF; Petrides, 2009); the Goldsmiths Musical Sophistication Index (Gold-MSI; Müllensiefen et al., 2014); the Mindset Scale (MS; Dweck and Leggett, 1988), the Grit Scale (GS; Duckworth and Quinn, 2009); the Social Skill Scale (SSS; Gismero, 2010); the Profile of Mood States (POMS; Butzer et al., 2016); the Patient Health Questionnaire (PHQ-9; Spitzer et al., 1999); the Beck Depression Inventory-II (BDI-2; Beck et al., 2009), the Self-Assessment Manikin questionnaire (SAMq), the Liebowitz Social Anxiety Scale (LSAS; Masia-Warner et al., 2003), the Music Performance Quality Scale (MPQ Scale; Nielsen et al., 2018), the Post-Music Performance Thoughts Questionnaire (PMPTQ; Nielsen et al., 2018), the Judge-rated Performance Quality (PQ; based on Montello et al., 1990), the Positive and Negative Affect Schedule (PANAS; Watson et al., 1988), the Music Emotional Contagion (MEC; Cohen and Bodner, 2019b), the Ten-Item Personality Inventory (TIPI; Gosling et al., 2003), the Internalized Shame Scale (ISS; Cook, 2001), the Multidimensional Perfectionism Scale (MPS; Hager and Flett, 2004), the Difficulties in Emotion Regulation Scale (DERS; Gratz and Roemer, 2004), the Positive Psychological Capital Questionnaire (PsyCap; Luthans et al., 2007), and the Rosenberg Self-Esteem Scale (RSES; Cai et al., 2009).

## 3 Results

The database search yielded 208 records, from which 31 duplicates were removed. Subsequently, 156 records were excluded for not meeting one or more of the predefined eligibility criteria, resulting in 21 studies selected for full-text review.

**Figure 1:**
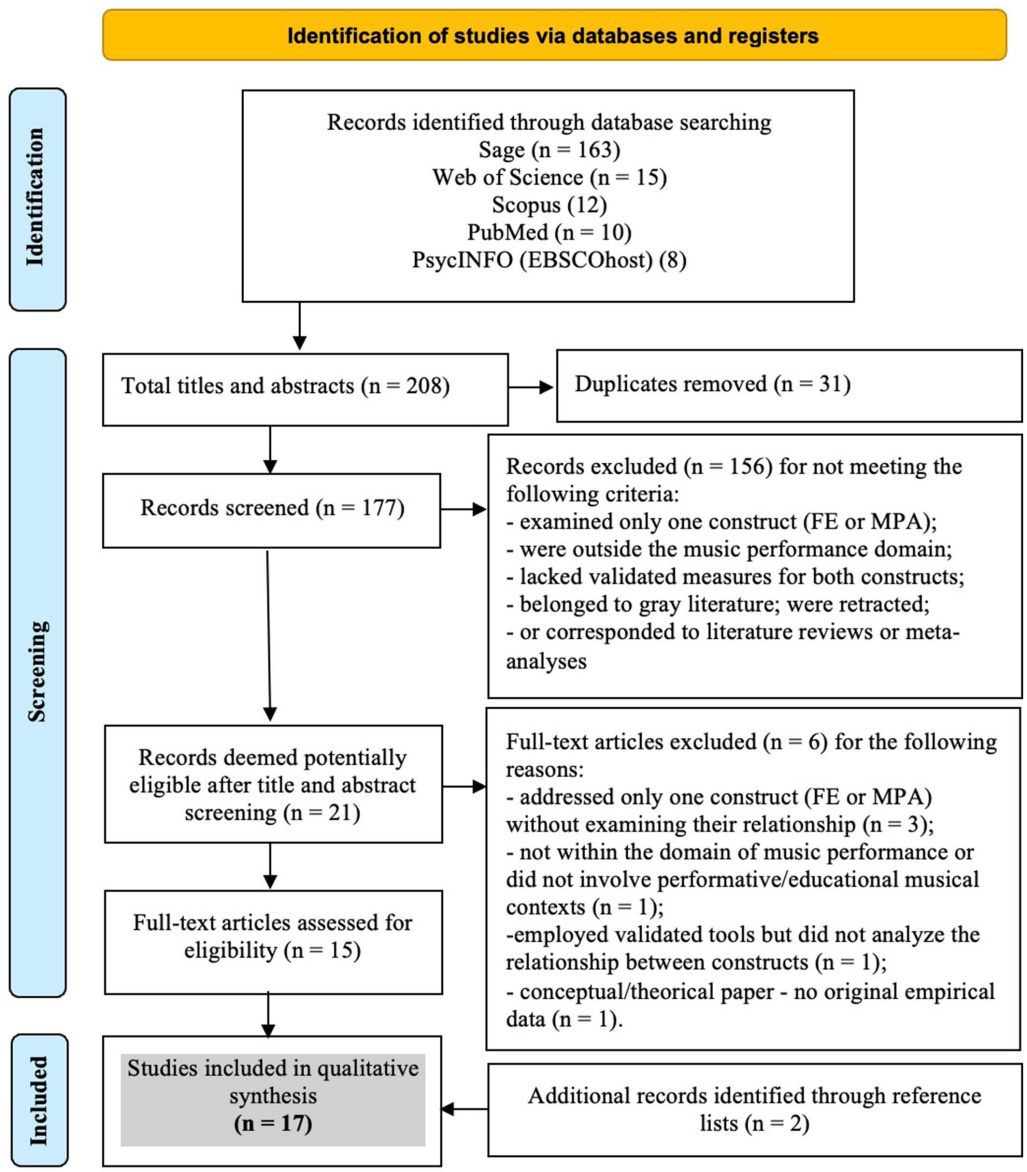
PRISMA flow diagram of study selection—a total of 17 studies were included after title and abstract screening, full-text assessment and eligibility verification.

After full-text screening, five studies were excluded for the following reasons: Moral-Bofill et al. (2023) mentioned a negative correlation between FE and MPA in the introduction but did not empirically measure or compare both constructs, focusing instead on predictors of flow; Bullerjahn et al. (2020) used tools assessing both FE and MPA but did not treat performance anxiety as a primary variable nor examine any direct relationship between the constructs; Carson and Collins (2016) and James et al. (1983) did not address the flow construct—conceptually or empirically—focusing exclusively on anxiety or physiological aspects of performance; Guyon et al. (2020a) included flow as a secondary variable without conducting direct analyses between FE and MPA. Following these exclusions, 15 studies were retained for analysis (see Figure 1) (Khalsa and Cope, 2006; Kirchner et al., 2008; Lamont, 2012; Fullagar et al., 2013; Butzer et al., 2016; Thomson et al., 2017; Cohen and Bodner, 2019a, 2019b, 2021; Spahn et al., 2021; Tan et al., 2021; Antonini Philippe et al., 2022b; Guyon et al., 2022; Jha et al., 2022; Moral-Bofill et al., 2022; Rakei et al., 2022; Jiang and Tong, 2024).

During the review of the included studies, two additional articles (Khalsa and Cope, 2006; Guyon et al., 2022) were identified and added, as they met all inclusion criteria. During the same process, Thurber et al. (2010) was initially considered for its conceptual discussion of the FE–MPA relationship and reference to the Flow State Scale used alongside anxiety measures. However, because it did not report empirical data on FE or examine the relationship between the constructs, it was ultimately excluded. Similarly, Kirchner (2011), which discusses the relationship between FE and MPA from a theoretical perspective, was excluded for being a purely conceptual paper without original empirical data. In total, 17 studies were included Figure 1 summarizes the selection process through the PRISMA flow diagram.

Table 1 summarizes the data of the 17 empirical studies included in this scoping review. To enhance comparative analysis and facilitate an integrated reading of the evidence, the table is structured into ten analytical categories: (1) “Author(s) and Year” identifies each publication chronologically and establishes the basis for cross-referencing with the review’s narrative synthesis; (2) “Main Aim” reports the primary research objective of each study; (3) “Sample Characteristics” details the sample examined, specifying the number of participants, gender distribution, level of expertise or professional status (students, teachers, or professional musicians), and country of origin; (4) “Measurement Instruments” lists the tools employed to assess FE and MPA (e.g., FSS, DFS-2, K-MPAI), including the type of measurement (state, trait, or mixed). (5) “Study Design” indicates the overall research design adopted (cross-sectional, qualitative, experimental, quasi-experimental, or longitudinal). (6) “FE–MPA Interaction” classifies the type of relationship investigated, distinguishing direct association, indirect, mediated or moderated effects, conceptual approaches, and psychophysiological examinations. (7) “Mediating / Moderating Factors” lists the variables examined as mediators or moderators of the FE–MPA relationship, with their corresponding measurement instruments reported within the same table column when applicable. (8) “Performance Setting” specifies the performance context analysed; (9) “Analytical or Procedural Approach” describes the analytical or procedural techniques employed; and (10) “Main Findings” summarizes the principal empirical outcomes of each study, highlighting how each study contributes to understanding the coexistence or interaction between flow and anxiety in performance. This structure enables a systematic overview across studies and clarifies the empirical, methodological, and contextual patterns through which the FE–MPA relationship has been explored.

Altogether, the contextual evidence demonstrates considerable heterogeneity in research designs, performance settings, and analytical approaches. The included studies range from controlled laboratory and simulated tasks to ecologically valid live performances, reflecting the methodological breadth through which the relationship between FE and MPA has been investigated.

Based on the analysis of Table 1, the presentation of the results was organized into seven thematic categories, with the aim of providing a clear and systematic structure for the diverse approaches and evidence identified across the included studies: (3.1) overview and evolution of research on the FE–MPA relationship; (3.2) sample characteristics and trends; (3.3) research design, performance contexts, and analytical approaches; (3.4) conceptual approaches and theoretical emphases; (3.5) interventional and regulatory perspectives; (3.6) measurement instruments and operational approaches; and (3.7) patterns of association between FE and MPA.

### 3.1 Overview and evolution of research on FE–MPA relationship

The first study to explicitly address the relationship between FE and MPA was conducted by Khalsa and Cope (2006), which investigated the regulatory potential of yoga-based practices as a self-regulation strategy in musical performance contexts, thereby establishing an early empirical framework for examining the interaction between flow-related processes and performance anxiety. Between 2006 and 2017, the literature progressively began to frame flow and anxiety as interdependent psychophysiological phenomena, emphasizing the emotional and cognitive components of the performance experience (Kirchner et al., 2008; Lamont, 2012; Fullagar et al., 2013; Butzer et al., 2016; Thomson et al., 2017). During this period, research primarily focused on describing how attention and self-efficacy (Kirchner et al., 2008), as well as emotional arousal and body–mind regulation (Lamont, 2012; Fullagar et al., 2013; Butzer et al., 2016; Thomson et al., 2017), interact in the modulation of musical performance.

From 2018 onwards, the field expanded conceptually and methodologically, with a new wave of studies examining the FE–MPA relationship through emotion-related, cognitive, and performance-related perspectives, rather than purely descriptive approaches (Cohen and Bodner, 2019a, 2019b, 2021; Spahn et al., 2021; Tan et al., 2021; Antonini Philippe et al., 2022a; Guyon et al., 2022; Jha et al., 2022; Moral-Bofill et al., 2022; Rakei et al., 2022; Jiang and Tong, 2024). Among the identified authors, Cohen and Bodner emerged as the most prolific, with three empirical publications (Cohen and Bodner, 2019a, 2019b, 2021) addressing FE and MPA. Within the emotional dimension, emotional intelligence and affective contagion were identified as mechanisms regulating the MPA-FE relationship. These factors influence emotional appraisal and sensitivity to evaluative cues during performance. Higher emotional intelligence and lower susceptibility to negative affective contagion were associated with lower MPA and higher FE (Cohen and Bodner, 2019b; Spahn et al., 2021; Tan et al., 2021; Antonini Philippe et al., 2022a). The cognitive dimension incorporated constructs such as self-regulation, self-efficacy, and metacognitive awareness. These variables support attentional control and adaptive coping, thereby facilitating flow and the management of anxiety during performance (Cohen and Bodner, 2019a; Jha et al., 2022; Jiang and Tong, 2024). At the level of situational influences, studies highlighted factors such as psychological capital, and performance conditions including audience presence and public versus private settings. For example, public performance situations were generally associated with increased MPA and reduced flow-related dimensions, although these patterns were not fully consistent across studies, suggesting sensitivity to task demands and performer characteristics (Guyon et al., 2022). Mindfulness was examined as an individual regulatory capacity influencing how performers respond to performance demands, operating alongside other emotional and cognitive variables (Tan et al., 2021; Moral-Bofill et al., 2022; Rakei et al., 2022). Collectively, this body of work reflects a transition toward a more integrative framework, in which flow and anxiety are conceptualized as co-regulated processes shaped by interacting emotional, cognitive, and performance-related mechanisms across diverse performance conditions.

### 3.2 Sample characteristics and trends

Sample sizes ranged from 11 to 664 participants, with the largest samples observed in cross-sectional studies (e.g., Rakei et al., 2022; Jiang and Tong, 2024) and the smallest in experimental and qualitative designs (e.g., Khalsa and Cope, 2006; Antonini Philippe et al., 2022b). Cross-sectional studies generally included more than one hundred participants, allowing broader correlational analyses, whereas intervention and longitudinal studies involved smaller, more controlled groups. Overall, gender distribution was balanced in most samples. However, some studies reported a female predominance, particularly among university and conservatory students (Lamont, 2012; Thomson et al., 2017), while Rakei et al. (2022), focusing on contemporary musicians, found a higher proportion of male participants.

The samples demonstrate a wide contextual diversity, ranging from professional and pre-professional musicians (Khalsa and Cope, 2006; Butzer et al., 2016; Thomson et al., 2017; Cohen and Bodner, 2019b, 2021; Tan et al., 2021) to university and conservatory students (Kirchner et al., 2008; Fullagar et al., 2013; Guyon et al., 2022; Jha et al., 2022; Moral-Bofill et al., 2022; Jiang and Tong, 2024). Most investigations focused on musicians in training, primarily university-level music students, but also psychology students (Lamont, 2012) and music therapy students (Cohen and Bodner, 2019a). Other studies involved conservatory teachers and students (Guyon et al., 2022; Moral-Bofill et al., 2022) as well as musicians at different professional levels (Antonini Philippe et al., 2022a).

Geographically, early research was concentrated in the United States (Khalsa and Cope, 2006; Kirchner et al., 2008; Fullagar et al., 2013; Butzer et al., 2016; Thomson et al., 2017) and the United Kingdom (Lamont, 2012). From 2018 onwards, research became more internationally distributed, with studies conducted in Israel (Cohen and Bodner, 2019a, 2019b, 2021), Germany (Spahn et al., 2021), Spain (Moral-Bofill et al., 2022), Switzerland (Antonini Philippe et al., 2022a; Guyon et al., 2022), Canada (Jha et al., 2022), China (Jiang and Tong, 2024), and multinational contexts (Rakei et al., 2022; Tan et al., 2021).

Overall, the sample data reveal a progressive diversification of participant profiles over the past two decades, from the early studies by small and relatively homogeneous samples (e.g., Khalsa and Cope, 2006) to more recent investigations involving larger and more heterogeneous samples (e.g., Jiang and Tong, 2024). The included studies involved musicians at different stages of training and professional development. Samples varied in terms of musical experience, age range, educational background, and geographical origin, indicating a broader range of participant profiles across the reviewed literature.

### 3.3 Research design, performance contexts, and analytical approaches

Five main types of research design were identified among the included studies. The majority consisted of cross-sectional studies, examining correlations between FE and MPA across varied performance contexts (Kirchner et al., 2008; Cohen and Bodner, 2019b, 2021; Spahn et al., 2021; Tan et al., 2021; Guyon et al., 2022; Jha et al., 2022; Rakei et al., 2022; Jiang and Tong, 2024). In addition, quasi-experimental and experimental studies explored the effects of intervention programs focused on yoga, mindfulness, self-regulation, or performance-based training (Khalsa and Cope, 2006; Butzer et al., 2016; Thomson et al., 2017; Cohen and Bodner, 2019a; Moral-Bofill et al., 2022). Qualitative studies analyzed musicians’ experiences through interviews, graphical representations, and narrative accounts (Lamont, 2012; Antonini Philippe et al., 2022a), and one longitudinal study followed participants over a seven-week period of musical practice and performance preparation (Fullagar et al., 2013).

The performance settings reported across the studies were diverse, encompassing real, educational or training-based, simulated, and laboratory environments. Real performance contexts were the most frequent, including concerts, exams, and rehearsals with student and professional musicians (Khalsa and Cope, 2006; Kirchner et al., 2008; Butzer et al., 2016; Cohen and Bodner, 2019b, 2021; Spahn et al., 2021; Moral-Bofill et al., 2022). Educational and training-based contexts were also common, particularly in studies implementing intervention programs such as yoga or self-regulation training conducted in conservatoires, universities, or summer courses (Khalsa and Cope, 2006; Butzer et al., 2016; Thomson et al., 2017; Guyon et al., 2022). Simulated environments were used in experimental and quasi-experimental designs to control contextual variables and reproduce performance-like conditions without audiences (Cohen and Bodner, 2019a; Tan et al., 2021). Laboratory studies were less frequent but allowed for physiological monitoring, such as heart rate variability (HRV) recordings during piano performance (Jha et al., 2022).

The analytical methods employed across the included studies were predominantly quantitative, involving correlation analyses, t-tests, regressions, and repeated-measures ANOVA to assess associations or pre–post intervention changes in FE and MPA. Mixed-design analyses compared public versus private performance conditions (Thomson et al., 2017), while structural equation modeling (SEM) was used to test relationships among psychological variables such as self-esteem, emotional intelligence, and flow (Jiang and Tong, 2024). Qualitative analytical procedures, including thematic or interpretative phenomenological analysis (IPA) to capture subjective experiences of flow and anxiety (Lamont, 2012; Antonini Philippe et al., 2022a). Some studies combined quantitative and qualitative approaches, integrating open-ended feedback or observational data to complement self-report measures (Cohen and Bodner, 2019b; Moral-Bofill et al., 2022).

### 3.4 Conceptual approaches and theoretical emphases

The conceptual dimension of the studies reviewed here shows a recurring focus on the relationship between FE and MPA, addressed from different conceptual approaches and theoretical emphases, including direct association frameworks, mediational and moderation models, and mechanism-oriented perspectives. Overall, the investigations examined the interaction between both constructs within educational and performative contexts, seeking to understand how they mutually influence each other—whether FE facilitates the control of MPA or, conversely, whether elevated anxiety levels restrict the propensity for FE.

Analysis of the nature of the relationship between FE and MPA reveals a predominance of studies adopting direct association approaches, primarily using correlational designs or pre–post comparisons to characterize FE–MPA associations, rather than to evaluate intervention efficacy (Khalsa and Cope, 2006; Kirchner et al., 2008; Fullagar et al., 2013; Butzer et al., 2016; Thomson et al., 2017; Cohen and Bodner, 2019b, 2021; Spahn et al., 2021; Guyon et al., 2022; Jha et al., 2022; Moral-Bofill et al., 2022). In contrast, a smaller subset of studies employed mediational or moderation models, representing an alternative conceptual focus aimed at identifying psychological and contextual mechanisms underlying FE–MPA associations. Among these, emotional factors such as musical emotional contagion (Cohen and Bodner, 2019a) and emotional intelligence (Rakei et al., 2022); motivational and cognitive variables such as grit and growth mindset (Tan et al., 2021), as well as psychological capital and self-esteem (Jiang and Tong, 2024); processes of self-regulation and social competence (Moral-Bofill et al., 2022); and contextual conditions such as audience presence (Guyon et al., 2022), were examined. Qualitative and interpretative approaches (Lamont, 2012; Antonini Philippe et al., 2022a) further contributed to this conceptual landscape by framing FE–MPA relations as experiential processes based on performers’ accounts. Collectively, these studies reflect a shift from describing associations to conceptually framing how specific mechanisms may shape FE–MPA interactions.

### 3.5 Interventional and applied perspectives

Among the intervention-based studies, two complementary orientations emerge regarding the direction of this interaction. The first assumes that reducing MPA facilitates the emergence of FE. Khalsa and Cope (2006) demonstrated that a yoga program conducted during a summer intensive course significantly reduced MPA, while Butzer et al. (2016), extending data collection over three consecutive years, confirmed that systematic yoga and meditation practice enhanced mindfulness and increased FE proneness. Similar results were reported by Cohen and Bodner (2019a) and Moral-Bofill et al. (2022), whose self-regulation training programs effectively decreased MPA and strengthened FE. The second orientation conceptualizes flow as a mechanism for MPA regulation, suggesting that flow mitigates MPA. Fullagar et al. (2013) found that progressive increases in FE during 7 weeks of performance practice led to reduced MPA; Thomson et al. (2017) observed a comparable pattern after intensive opera training; Tan et al. (2021) showed that motivational traits such as grit and growth mindset predicted higher flow and lower MPA; and Jiang and Tong (2024) identified flow and self-esteem as mediators in the association between psychological capital to MPA reduction. Together, these studies demonstrate that self-regulation and flow operate as complementary mechanisms in the modulation of emotional states during musical performance.

### 3.6 Measurement instruments and operational approaches

As summarized in Table 1, the operationalization of FE and MPA across the analyzed studies relied predominantly on a relatively small set of internationally validated self-report instruments, consistently applied in musical performance contexts. FE was operationalized as a multidimensional experiential construct, grounded in the theoretical flow framework and its nine core dimensions as described in the Introduction, and was most assessed using the FSS, the DFS-2, and music-specific adaptations derived from the same dimensional framework, such as the EFIM and the MFS. In contrast, MPA was operationalized as performance-related anxiety, primarily measured using music-focused instruments, such as the PAI and the K-MPAI, and, in some studies, through general anxiety measures, notably the STAI, when explicitly applied to performance situations. Together, these instruments represent the most frequently adopted approaches for operationalizing FE and MPA in the reviewed literature.

Some studies examined cross-interactions between dispositional and situational measures, including correlations between dispositional FE and state MPA (Cohen and Bodner, 2019b, 2021; Tan et al., 2021; Rakei et al., 2022) and between situational FE and trait MPA (Spahn et al., 2021; Moral-Bofill et al., 2022). Complementarily, other studies approached the FE–MPA relationship through pre–post program evaluations (Khalsa et al., 2013; Butzer et al., 2016; Thomson et al., 2017; Cohen and Bodner, 2019a), qualitative analyses of experiential coexistence (Lamont, 2012; Antonini Philippe et al., 2022a), or mediated structural models (Jiang and Tong, 2024). These studies consistently reported inverse associations between the constructs, despite differences in measurement level and temporal framing. Across studies, the FE and MPA scales were applied to performance contexts, with some authors reporting psychometric or contextual limitations when used in musical settings. In some cases, adapted or partial versions of the original instruments were used, with terminological or format adjustments to better reflect the characteristics of musical performance (Kirchner et al., 2008; Guyon et al., 2022; Moral-Bofill et al., 2022). Additional approaches incorporated psychophysiological indicators, such as HRV, to examine relationships between physiological parameters, levels of FE, and MPA during performance tasks (Jha et al., 2022). Moreover, longitudinal and experimental studies adopted different temporal designs: Fullagar et al. (2013) monitored changes in FE and MPA across several weeks of performance practice, whereas Spahn et al. (2021) collected data before, during, and after performance, allowing the temporal dynamics of these states to be captured. Collectively, the reported evidence describes a diversity of methodological approaches, combining dispositional, situational, and physiological measures in the analysis of the relationship between FE and MPA.

### 3.7 Patterns of association between FE and MPA

Overall, the empirical studies identified a negative relationship between FE and MPA, observed across different contexts, samples, and methodological designs (Khalsa and Cope, 2006; Kirchner et al., 2008; Butzer et al., 2016; Thomson et al., 2017; Cohen and Bodner, 2019a, 2019b, 2021; Spahn et al., 2021; Tan et al., 2021; Guyon et al., 2022; Moral-Bofill et al., 2022; Rakei et al., 2022; Jiang and Tong, 2024). An inverse association was observed across studies, with stronger correlations typically reported when state-based measures were used and when data were collected in real or simulated performance contexts, compared with trait-based assessments and laboratory tasks. Correlations tended to be stronger in real or simulated performance situations than in laboratory tasks. Laboratory-based evidence incorporating physiological indicators, such as heart rate variability (HRV), aligned with this pattern by characterizing associations between FE, autonomic regulation, and MPA (Jha et al., 2022).

In addition to inverse associations, qualitative and interpretative studies (Lamont, 2012; Antonini Philippe et al., 2022a) identified patterns of coexistence of FE and MPA, particularly under conditions of high challenge or perceived demand. In these studies, participants described experiences of simultaneous anxiety and high concentration during performance. These patterns indicated that FE and MPA are not mutually exclusive states, and may be reported concurrently under conditions of high challenge or perceived demand. Taken together, the results show that the relationship between FE and MPA was examined through diverse methodological approaches—cross-sectional, experimental, and qualitative—that explored multiple levels of interaction between the two constructs.

## 4 Discussion

This scoping review synthesized the empirical and theoretical evidence examining how FE and MPA have been examined together, measured, and interpreted in performative and educational contexts. The analysis of 17 studies published over the past two decades revealed a predominantly negative relationship between the two constructs, observed consistently across populations, methodologies, and performance settings. However, several studies also indicated that moderate and adaptive levels of MPA may coexist with or even facilitate flow when appraised as a challenge rather than a threat. Thus, the results of this review fulfill its primary objective by providing an integrative overview of how both experiences have been jointly studied, highlighting conceptual tendencies, methodological diversity, and empirical gaps that may guide future research and inform the design of interventions aimed at fostering optimal engagement and emotional regulation among musicians. The following sections discuss these findings from conceptual, methodological, and applied perspectives.

The analysis of samples revealed substantial variation in size and profile, ranging from small groups of students to large cohorts of hundreds of participants, which limits generalizability. This evolution reflects an increasingly contextualized and ecological approach to studying the performance experience. Such diversification has enabled the identification of psychological and developmental variables associated with flow propensity—such as self-efficacy, self-compassion, and emotional intelligence (Tan et al., 2021; Guyon et al., 2022)—consistent with recent literature on motivation and emotional regulation in music learning (Miksza and Tan, 2015; Biasutti and Habe, 2023; Habe and Biasutti, 2024). From a methodological perspective, this heterogeneity invites a cautious and nuanced interpretation of findings across studies conducted in different educational and professional settings (Arksey and O’Malley, 2005; Levac et al., 2010). It also points to the value of greater conceptual and methodological transparency when synthesizing evidence across contexts, particularly regarding how key analytical dimensions are framed and operationalized in scoping reviews (Peters et al., 2020).

At the theoretical level, supported by empirical evidence, the analysis reveals a consistent negative correlation between FE and MPA, observed in both dispositional and situational measures. This pattern aligns with Csikszentmihalyi’s classic model (Csikszentmihalyi 1975, 1990), which posits that flow arises from the balance between challenge and skill, while an imbalance favoring challenge produces anxiety. Some core phenomenological dimensions of flow—particularly task absorption, reduced self-consciousness, and a sense of control as described by Nakamura and Csikszentmihalyi (2009) are theoretically incompatible with the hypervigilance, threat monitoring, and evaluative fear that characterize MPA within cognitive models (Eysenck and Calvo, 1992; Kenny, 2011). This incompatibility is illustrated by Kirchner (2011) through the contrast of paired experiential features of FE and MPA, such as absorption in the task versus distraction, process-oriented versus outcome-focused goals, a sense of control versus fear of losing control, and reduced versus heightened self-consciousness. This incompatibility is supported by empirical findings showing that high MPA levels reduce attentional focus (Matei and Ginsborg, 2017; Oudejans et al., 2017) and flow propensity (Spahn et al., 2021; Guyon et al., 2022).

However, recent evidence suggests a more dynamic rather than dichotomous relationship between FE and MPA, indicating that both experiences can coexist and interact within the same performance episode (Lamont, 2012; Cohen and Bodner, 2019b, 2021; Spahn et al., 2021; Tan et al., 2021; Antonini Philippe et al., 2022a). Contemporary perspectives distinguish adaptive from maladaptive forms of MPA (Papageorgi et al., 2013; Papageorgi, 2021, 2022), in accordance with the classic inverted-U law of Yerkes and Dodson (1908): moderate emotional activation, interpreted as challenge, may enhance concentration and control, whereas excessive activation, perceived as threat, triggers rumination and performance impairment (Osborne and Kenny, 2008; Kenny, 2011; Papageorgi, 2021; Herman and Clark, 2023; Habe and Biasutti, 2024). Recent empirical evidence further indicates that MPA is not inherently detrimental but may exert either facilitating or debilitating effects on performance depending on its intensity, appraisal, and regulatory processes (Murphy et al., 2025). Accordingly, the relationship between MPA and FE should be understood as a dynamic continuum in which the performer’s emotional regulation determines whether MPA functions as a facilitator or inhibitor of optimal engagement (Papageorgi et al., 2007; Papageorgi et al., 2013; Papageorgi, 2021). Qualitative evidence supports this view, showing that initial arousal and moderate MPA can serve as functional triggers for FE, reflecting a gradual transition from tension to full absorption (Lamont, 2012; Antonini Philippe et al., 2022a). This dialectical view is further reinforced by Cohen and Bodner (2021), Spahn et al. (2021), and Tan et al. (2021), who argue that the goal is not to eliminate MPA but to transform it into functional activation that enhances performance.

Cross-sectional studies stand out not only for their numerical prevalence but also for their recency—seven were published between 2021 and 2024. These studies tested correlations between FE and MPA across diverse performance settings (e.g., orchestral and solo), identifying factors that promote flow and, consequently, anxiety control. Such factors include contextual (e.g., orchestral role, instrument, performance context) and background variables (e.g., gender, age, culture) (Cohen and Bodner, 2021); musical training and perseverance (Tan et al., 2021); age, professional experience, performance importance, and self-efficacy (Spahn et al., 2021) the influence of audience presence (Guyon et al., 2022); locus of control, courage, openness, conscientiousness, and emotional stability (Rakei et al., 2022); and lower sympathetic activation before performance (Jha et al., 2022). More recently, Jiang and Tong (2024) proposed a mediational model in which psychological capital and self-esteem influence anxiety and flow, suggesting that strengthening internal psychological resources may simultaneously reduce MPA and enhance FE, advancing the understanding of affective and cognitive mediation mechanisms between the two phenomena.

Methodologically, research has evolved from predominantly correlational designs toward mediational and integrative approaches. Although interventions such as Kripalu yoga (Khalsa and Cope, 2006; Butzer et al., 2016), intensive opera training (Thomson et al., 2017), mental and physiological awareness training (Cohen and Bodner, 2019a), and psychological self-regulation programs (Moral-Bofill et al., 2022) have proven effective in managing MPA and facilitating FE, determining which strategies are most effective falls beyond the scope of this review, as current evidence remains inconclusive. These results align with positive psychology (Seligman, 2008; Seligman and Csikszentmihalyi, 2014), emphasizing that interventions fostering well-being and self-control help reduce anxiety and promote flow.

The number of experimental studies remains limited, and, as Cohen and Bodner (2021) highlight, longitudinal research is still needed to map the temporal progression of MPA and FE. Moreover, it is necessary to explore different strategies that could define consistent pathways for promoting flow and managing anxiety. Flow theory enhances understanding of musical engagement and provides pedagogical guidance (Fullagar et al., 2013). Recognized as a positive performance state, it encourages educators to foster it among students (Sinnamon et al., 2012). In classical music students, FE enhance post-performance confidence and coping with MPA (Clark et al., 2014; Iusca, 2015; Spahn et al., 2021).

The diversity of study designs observed across the reviewed literature has important implications for how the relationship between FE and MPA has been interpreted. Intervention-based and qualitative studies typically relied on smaller samples, often composed of young or student musicians in educational contexts, which favored methodological control and participant engagement but limited generalizability (Khalsa and Cope, 2006; Kirchner et al., 2008; Lamont, 2012; Fullagar et al., 2013; Butzer et al., 2016; Cohen and Bodner, 2019a; Antonini Philippe et al., 2022a). These designs were particularly useful for testing training-based interventions aimed at enhancing flow or managing anxiety, allowing a closer observation of intraindividual changes during performance. In contrast, cross-sectional studies—more numerous and statistically robust—examined larger and more heterogeneous samples of experienced or professional musicians (Cohen and Bodner, 2019b, 2021; Spahn et al., 2021; Tan et al., 2021; Rakei et al., 2022; Jiang and Tong, 2024), offering broader evidence of the general trends linking flow and anxiety but less sensitivity to individual or situational nuances.

This methodological divide suggests that intervention and correlational approaches capture different aspects of the same phenomenon: the former illustrate how regulatory strategies may promote FE and reduce MPA, while the latter identify which psychological traits or contextual variables predict these states. Evidence from both approaches converges on the notion that musical experience level and performance context critically shape this dynamic. While greater age and expertise have been associated with more frequent or stable experiences of FE (Spahn et al., 2021), studies also indicate that professional musicians may experience constraints on flow when high self-imposed performance standards and expectations are present (Butzer et al., 2016). Conversely, musicians in training demonstrate greater responsiveness to self-regulation and emotional learning strategies, making them a key sample for preventive interventions (Braden et al., 2015; Nwokenna et al., 2022).

Several studies further indicate that stylistic diversity and musical background influence how performers experience the challenge–skill balance. Kirchner et al. (2008) noted that university students may require more time to achieve flow compared with professionals, while Tan et al. (2021) and Rakei et al. (2022) demonstrated that non-classical and mixed-genre musicians display distinctive flow–anxiety patterns, such as greater tolerance of arousal and more flexible attentional focus during performance, possibly reflecting different motivational and cultural frameworks (e.g., emphasis on expressivity, improvisation, and intrinsic motivation rather than error avoidance and evaluative precision). These findings underscore that flow and MPA are not merely universal constructs but are modulated by artistic identity and learning environment. Therefore, future investigations should continue to expand the ecological scope of sampling, integrating musicians from varied genres, experience levels, and cultural contexts to capture the full range of regulatory and experiential mechanisms underpinning the FE–MPA relationship.

Methodological limitations were observed at two levels: (1) organizational—lack of control groups for simulated performances (Cohen and Bodner, 2019a), total absence of controls (Thomson et al., 2017), logistical constraints (Khalsa and Cope, 2006), random group allocation affecting comparability (Butzer et al., 2016), and artificiality of laboratory performance contexts (Guyon et al., 2022); and, (2) motivational—high expectations of improvement may bias perceived benefits (Butzer et al., 2016). Cohen and Bodner (2019a), applying the short version of the DFS-2, reported acceptable reliability across eight of nine dimensions but had to exclude the action–awareness fusion item due to low internal consistency, consistent with findings from Sinnamon et al. (2012) and Wrigley and Emmerson (2013). Although Jackson and Csikszentmihalyi (1999) regarded this fusion as essential in sports contexts, it may not be central in musical performance. Accordingly, Cohen and Bodner (2019b, 2021) and Fullagar et al. (2013) called for the development of music-specific flow measures. Subsequent authors (Guyon et al., 2022; Tan et al., 2021) reinforced the need to assess all nine flow dimensions in the musical domain. Fullagar et al. (2013) applied brief scales to assess FE, perceived challenge, skill, and anxiety, though without addressing psychometric validity. Thus, future research should prioritize the creation and validation of domain-specific FE measures for musical performance.

Corroborating the present findings, Moral-Bofill et al. (2023) demonstrated that positive self-assessment of musical competence is a key predictor of FE, emphasizing the importance of confidence in one’s abilities. The presence of significant musical challenges also promoted flow, suggesting that task complexity can enhance optimal engagement. Intrinsic motivation and goal clarity further contributed to FE, while higher anxiety levels hindered it. These results shed light on the psychological determinants of flow in musicians, shifting research focus from anxiety reduction toward strategies that actively foster flow.

A further issue concerns the operationalization of flow as an optimal and intrinsically rewarding condition—crucial for exceptional performance and sustained engagement (Csikszentmihalyi, 1988). Flow functions as a source of intrinsic motivation in complex, attention-demanding tasks. Abuhamdeh (2020) proposed conceptualizing flow as a discrete construct; however, nonlinear regression analyses reveal sudden changes in FE, emphasizing its presence–absence nature (Bricteux et al., 2017). Critiques of widely used scales such as the FSS-2 point to limitations, particularly their neglect of the condition–experience model (Moneta, 2021). Adaptations integrating this model in musical contexts face challenges concerning scale sensitivity and threshold definitions for identifying the flow state (Moral-Bofill et al., 2022). Thomson et al. (2017) reported difficulties with self-report measures and found no statistically significant differences between a short-term (2-week) and a longer-term (4-week) intensive opera training program. Khalsa and Cope (2006) similarly failed to obtain statistically significant changes in flow scores following the yoga-based intervention, possibly due to participants’ high expertise. The absence of control groups in Khalsa and Cope (2006) and Butzer et al. (2016) further constrained the internal validity of their studies. Demographic limitations in Cohen and Bodner’s studies hindered control over cultural factors in Israeli orchestras, while the cross-sectional design of Cohen and Bodner (2021) limited trait-level interpretation of FE–MPA dynamics. These issues call for cautious interpretation of results.

Overall, the interaction between MPA and FE remains a promising yet challenging field of inquiry. Individual variability emerges as a key determinant, as responses to anxiety and flow differ markedly between musicians: stimuli that provoke anxiety in some may trigger flow in others. Conceptual inconsistencies across studies complicate direct comparisons, underscoring the need for standardized definitions to ensure result comparability. Performance context also plays a critical role, as live and studio performances evoke distinct emotional dynamics. External factors such as environment, audience, and familiarity with repertoire influence both anxiety and flow, complicating experimental control. The frequent reliance on self-reports introduces subjectivity and limits objectivity. Moreover, the absence of formal quality appraisal or risk-of-bias assessment—consistent with the scope and aims of scoping review methodologies—limits the possibility of weighing the relative strength of the available evidence. While most studies examine immediate correlations, the lack of longitudinal research prevents understanding of the temporal evolution of FE–MPA interactions. Establishing causal relationships is equally complex, as MPA may hinder FE, yet effective FE can mitigate anxiety. Addressing these methodological and conceptual challenges will refine future investigations and deepen understanding of this interplay.

Despite these limitations, the identified gaps provide valuable guidance for future research. Multidisciplinary approaches acknowledging the complexity of these phenomena may enrich the field and promote significant advances. The gaps identified and the perspectives outlined in this article suggest that future research should prioritize psychophysiological monitoring as an empirical basis for understanding FE–MPA relationships, include larger and more diverse samples, and consider trait levels of both constructs as baselines. In addition, reliance on the published literature may have favored studies reporting clearer or more readily interpretable patterns, potentially underrepresenting less conclusive or exploratory findings, highlighting the need for greater methodological transparency and consistency in future work. Longitudinal, mixed-method, and multimodal intervention designs may represent promising pathways for fostering flow propensity and adaptive anxiety regulation in musicians.
